# Gut Microbial Diversity Analysis of Different Native Chickens and Screening of Chicken-Derived Probiotics

**DOI:** 10.3390/ani13233672

**Published:** 2023-11-27

**Authors:** Lu Chen, Xue Bai, Tao Wang, Jia Liu, Xiaomeng Miao, Bo Zeng, Diyan Li

**Affiliations:** 1 School of Pharmacy, Chengdu University, Chengdu 610106, China; lc628633@163.com (L.C.); wanttao3@cdu.edu.cn (T.W.); 2 College of Animal Science and Technology, Sichuan Agricultural University, Chengdu 611130, China; 18792972819@163.com; 3 Guizhou Province Livestock and Poultry Genetic Resources Management Station, Guiyang 550001, China; liujia994@126.com; 4 Institute of Animal Husbandry and Veterinary Medicine, Guizhou Academy of Agricultural Sciences, Guiyang 550005, China; mxm19920404@126.com

**Keywords:** native chicken, gut microbes, 16S rRNA, bacterial isolation and screening, whole genome sequencing

## Abstract

**Simple Summary:**

The gut microbiota plays a vital role in the growth and development of chickens, while environmental and host factors can influence its composition. This study sheds light on the variations in gut microbial diversity and structural composition among native chickens from different geographical environments, as well as different breeds of broiler and laying hens, revealing the presence of distinct microbial communities. Additionally, our investigation into chicken-derived probiotics, specifically *Lactobacillus agilis* MH1 and *Lactobacillus salivarius* ZJ1, uncovered their probiotic properties, favorable genomic profiles, and their ability to alleviate enteritis in mice. These findings provide a critical theoretical foundation for the study of native chickens and offer valuable guidance for the subsequent development and formulation of chicken-derived probiotics.

**Abstract:**

The gut microbiota plays a critical role in the growth, development, nutritional digestion, and overall health of chickens. Furthermore, certain probiotics isolated from poultry intestines have demonstrated the potential to enhance immune function and production performance in chickens. To investigate the differences in gut microbiota among chickens from various geographical environments and different breeds of broiler and laying hens, we conducted 16S rRNA sequencing on the fecal microbiota of 140 Chinese native chickens and ten Roman layers. In addition, we isolated and screened the potential probiotics to examine their biological characteristics, genome profiles, and functionality in animals. Our findings revealed the significant variations in gut microbiota composition and structure between Tibetan chickens (ZJ), which reside in high-altitude regions, and Meihua chickens (MH) and Xuhai chickens (XH), which inhabit low-altitude regions. Specifically, *Cupriavidus* and *Candidatus_Bacilloplasma* were identified as unique microbial communities in high and low altitudes, respectively. Notably, among regions with similar altitudes, Luning chickens (LN) exhibited the lowest α diversity, accompanied by a remarkably high relative abundance of Firmicutes and Lactobacillus. Conversely, Wugu chickens (WGs) and Yaoshan chickens (YSs) displayed similar gut microbiota profiles. Furthermore, distinctive gut microbiota patterns were observed between the different breeds of broilers and laying hens. Commercial Roman layers (LMs) exhibited significantly lower alpha diversity compared to native chickens, and broilers and laying hens predominantly harbored Firmicutes, Bacteroidota, and Proteobacteria. Of particular interest, the probiotics *Lactobacillus agilis* MH1 and *Lactobacillus salivarius* ZJ1, derived from chicken feces, exhibited favorable genomic profiles, and demonstrated anti-colitis effects and immunomodulatory functions. These findings provide a crucial theoretical foundation for native chicken research and offer insights for the future development and formulation of chicken-derived probiotics.

## 1. Introduction

The structure and function of gut microbes play a crucial role in poultry health, and the acquisition and maturation of gut microbiota throughout the entire growth cycle of chickens have profound effects on growth and development [1], gut health [2,3], and nutritional digestion [4,5]. Symbiotic bacteria in the digestive system, for instance, contribute vital nutrients to poultry metabolism, including short-chain fatty acids, ammonia, amino acids, and vitamins [6,7,8,9]. Moreover, the gut microbiome is involved in immune regulation [10,11]. Previous studies have identified bacterial groups associated with chicken immunity, such as the positive correlation between an increase in Proteobacteria and pro-inflammatory cytokines, as well as the positive correlation between an increase in Firmicutes and the expression of the anti-inflammatory factor *TGF-β4* [12,13]. Additionally, the impact of gut microbiota on chicken performance is evident in body weight [14,15,16], feed conversion rate [17], egg production [18], and meat quality [19].

In the poultry industry, meat and eggs are essential components of daily life. Native chicken varieties, in particular, are highly favored due to their adaptability, flavorful meat, and nutritional richness. Each native chicken breed represents an excellent variety bred in specific geographical environments (topography, climate) over an extended period [20]. Numerous studies have demonstrated variations in gut microorganisms among chickens from different geographical environments, with the geographical location playing a prominent role in shaping the chicken gut microflora [21]. These variations are influenced by factors such as altitude [22], temperature [23], and diet [24]. Additionally, the genetic background of chickens within the same breeding environment also influences their gut microbiota. Different breeds of broilers, for example, exhibit distinct ileum microbiota [25]. Qi et al. [26] and Willson et al. [27] have reported significant differences in the microbial composition between broilers and laying hens.

In recent years, probiotics have emerged as alternatives to antibiotics in poultry production. They can promote the growth of beneficial bacteria, inhibit the growth of pathogenic bacteria [28], produce various digestive enzymes, and improve immunomodulatory molecules to maintain a balanced gut flora in livestock and poultry. This, in turn, reduces gut diseases, enhances feed digestion and absorption, improves immune function, and enhances the overall performance [29,30,31,32,33]. *Lactobacillus* and *Bacillus*, in particular, have demonstrated efficacy in poultry. For instance, the application of *Lactobacillus rhamnosus* in poultry significantly reduced the diarrhea rate in chickens infected with *Salmonella typhimurium* [34], while *Bacillus* subtilis was found to reduce the colonization of *Campylobacter jejuni* and increase weight gain [35].

Building upon the aforementioned findings, this study aims to explore the diversity and composition of gut microbiota in different native chicken breeds. By analyzing the gut microorganisms of native chickens from various geographical environments and different broiler and laying hen breeds, we aim to identify the unique gut flora associated with native chickens. The study will provide insights into the relationship between gut microbes, environmental adaptations, and breeds. Additionally, we will isolate and screen the probiotics derived from native chickens and assess their functional properties. These findings will establish a crucial theoretical foundation for native chicken research and serve as a reference for the subsequent development and formulation of chicken-derived probiotics.

## 2. Materials and Methods

### 2.1. Experimental Chicken

**Figure 1:**
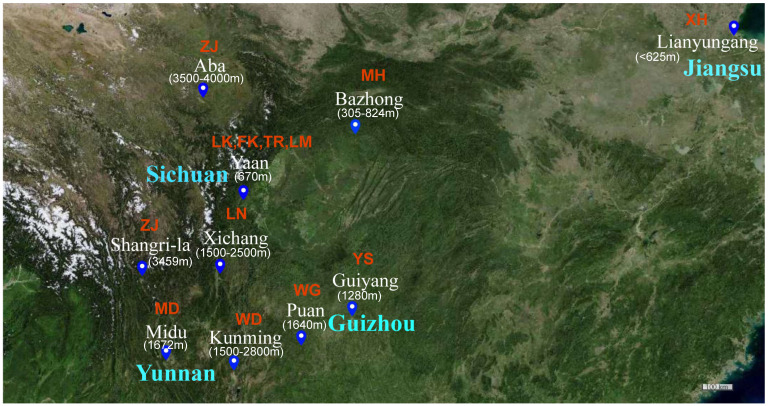
Sample collection site of native chickens. ZJ: Tibetan Chicken, MH: Mountainous Meihua Chicken, XH: Xuhai Chicken, MD: Midu Chicken, WD: Wuding Chicken, LN: Luning Chicken, WG: Black-bone Chicken, YS: Yaoshan Chicken, LK: Tianfu green shell layer, FK: Tianfu powder shell laying chicken, LM: Roman pink shell layer, TR: Tianfu broiler.

A total of 110 fecal samples were collected from free-range native chicken breeds at approximately 52 weeks of age (peak laying period) in Yunnan (Midu Chicken (MD, *n* = 10), Wuding Chicken (WD, *n* = 10), Tibetan Chicken (ZJ, *n* = 10)), Guizhou (Black-bone Chicken (WG, *n* = 20), Yaoshan Chicken (YS, *n* = 20)), Sichuan (Tibetan Chicken (ZJ, *n* = 10), Mountainous Meihua Chicken (MH, *n* = 10), Luning Chicken (LN, *n* = 10)) and Jiangsu (Xuhai Chicken (XH, *n* = 10)) provinces, maintaining a sex ratio of 1:1. These chickens represent the main southwest China at different altitudes and East China areas. Additionally, we collected 40 fecal samples from three 43-week-old (peak laying period) laying hens (Tianfu green shell layer (LK, *n* = 10), Tianfu powder shell laying chicken (FK, *n* = 10), Roman layer (LM, *n* = 10)) and one Tianfu broiler (TR, *n* = 10) at the poultry breeding base of Sichuan Agricultural University in Ya’an, Sichuan, with a sample size of 10 for each breed (Figure 1, Table S1). Fresh fecal samples weighing 2 g were collected using cotton swabs, transferred to 2 mL sterile EP tubes, immediately frozen in liquid nitrogen and subsequently stored in a laboratory freezer at −80 °C for DNA extraction and bacterial isolation.

### 2.2. DNA Extraction and 16S rRNA Sequencing Analysis

Fecal microbial DNA was extracted using the TIANamp Stool DNA Kit (Tiangen, Beijing, China) following the manufacturer’s instructions. The quality and integrity of the extracted DNA were assessed using a NanoDrop 2000 spectrophotometer and 1% agarose gel electrophoresis. The V3–V4 hypervariable regions of the bacterial 16S rRNA gene were amplified using the forward primer 341F (5′-CCTAYGGGRBGCASCAG-3′) and reverse primer 806R (5′-GGACTACNNGGGTATCTAAT-3′) [36]. The PCR reaction system consisted of 15 μL Phusion^®^ High-Fidelity PCR Master Mix (New England Biolabs, Beijing, China), 0.2 μM forward and reverse primers, and approximately 10 ng of template DNA. The PCR reaction cycle included an initial denaturation at 98 °C for 1 min, followed by 30 cycles of denaturation at 98 °C for 10 s, annealing at 50 °C for 30 s, elongation at 72 °C for 30 s, and a final extension at 72 °C for 5 min. PCR products were purified using the Qiagen Gel Extraction Kit (Qiagen, Hilden, Germany), and the TruSeq^®^ DNA PCR-Free Sample Preparation Kit was used to generate sequencing libraries. The constructed libraries were quantified using Qubit@2.0 and evaluated using the Agilent Bioanalyzer 2100 system. Finally, the libraries were sequenced on the Illumina NovaSeq platform to generate 250 bp paired-end reads.

The raw 16S rRNA gene sequencing data were processed using QIIME2 [37]. Adapters and low-quality reads were removed from the data to obtain clean reads. The clean reads were then clustered to obtain amplicon sequence variants (ASVs). High-quality representative feature sequences were used as references for taxonomic annotation using the Silva database (138_99 release) (https://www.arb-silva.de/ (accessed on 5 May 2022)). The richness and evenness of the microbial community were measured using the observed features and Shannon index, and the Kruskal–Wallis H test was used to compare the different groups. β-diversity analysis, based on the Bray–Curtis distance matrix and Jaccard distance matrix, was used to assess the similarity of microbial composition between samples through principal coordinate analysis (PCoA) and the permutation analysis of variance (PERMANOVA). Linear discriminant analysis (LDA) was performed to identify significant differences in gut microbiota between chickens from different locations (LDA > 3, *p* < 0.05).

### 2.3. Isolation and Identification of Probiotics

Native chicken fecal samples were added to the PBS buffer at a 1:10 ratio and thoroughly shaken and mixed. The mixture was divided into two tubes. One tube underwent serial dilution from 10^−1^ to 10^−5^, and 100 μL of each dilution was plated on de Man, Rogosa, and Sharpe agar (MRS) and anaerobically incubated at 37 °C for 48 h. The other tube was subjected to heat treatment at 80 °C for 10 min, and the supernatant (100 μL) was plated on nutrient agar (NA) and aerobically incubated at 37 °C for 48 h. Colonies with different shapes and sizes were selected and passaged three times on a new culture medium to obtain pure strains. The further identification of bacterial species was conducted using 16S rRNA sequencing. The strain DNA was extracted, and PCR amplification was performed using universal primers 27F (5′-AGAGTTTGATCCTGGCTCAG-3′) and 1492R (5′-GGTTACCTTGTTACGACTT-3′) [38]. The PCR products were sequenced by Sangon Biotech Company (Shanghai, China), and the sequencing results were compared with the BLAST in the NCBI database for bacterial classification.

For the acid *production assay*, *Lactobacillus* strains were activated and resuspended in sterile PBS buffer, and the OD600 was adjusted to 1 using an enzyme marker. The bacterial suspension was inoculated in MRS broth at a 5% concentration and anaerobically incubated at 37 °C for 24 h. MRS broth without bacteria served as the control group, and pH values were measured at 2 h, 6 h, 12 h, and 24 h during the incubation.

For the enzyme production assay, three 6 mm sterile filter papers were placed on a soluble starch medium and a casein hydrolysis medium. Activated *Bacillus* suspension (10 μL) was added dropwise onto the filter papers, while filter papers without bacteria solution served as the control. After incubation at 37 °C for 24 h, the presence of hydrolysis circles was observed, and the diameter of the circles was measured after staining the soluble starch plates with iodine solution.

For the hydrophobicity assay [39], the isolated strains were grown overnight, and the OD600 values were adjusted to approximately 0.5 (A0) after two resuspension washes. A test tube containing 1 mL of xylene was mixed with 3 mL of the strain suspension and vortexed for 1 min. The mixture was then incubated at 37 °C for 2 h, and the optical density of the aqueous phase at 600 nm (A2) was measured. This experiment was repeated three times, and strain hydrophobicity was calculated using the formula: (A0 − A2)/A0 × 100%.

For the auto-aggregation assay [39], the isolated strains were grown overnight, and the OD600 values were adjusted to approximately 0.5 (A0) after two resuspension washes. A bacterial suspension of 4 mL was transferred to a new centrifuge tube and incubated at 37 °C for 24 h. The upper layer of liquid was then collected to determine the OD600 value (A24), and the experiment was repeated three times. The strain auto-aggregation ability was calculated using the formula: (A0 − A24)/A0 × 100%.

The Kirby–Bauer method (K-B) was employed to assess the antibiotic sensitivity of the isolated strains [40]. *Escherichia coli* ATCC25922 and *Staphylococcus aureus* ATCC25923 were used as the quality control strains. Bacterial fluid with a 0.5 McFarland standard was evenly spread onto MRS or NA agar plates using a sterile cotton swab. After drying for 3–5 min, antibiotic drug patches (10 µg ampicillin, 30 µg cefotaxime, 10 units of penicillin, 30 µg amikacin, 10 µg streptomycin, 10 µg gentamicin, 10 µg norfloxacin, 5 µg ciprofloxacin, 5 µg levofloxacin, 15 µg erythromycin, 30 µg tetracycline, 30 µg vancomycin, 30 µg chloramphenicol, and 2 µg clindamycin) were evenly placed on the plate surface using sterile forceps clips. The plates were then incubated at 37 °C for 24 h, and the diameter of the inhibition zone was measured using a vernier caliper. The test results were analyzed according to the most recent criteria provided by the Clinical and Laboratory Standards Institute (CLSI).

The Oxford cup method was used to determine the antibacterial ability of the isolated probiotics against pathogenic bacteria [41]. The indicator pathogenic bacteria included *Escherichia coli* ATCC25922, *Staphylococcus aureus* ATCC25923, and *Salmonella enterica subsp. enterica serovar* ATCC14028. The concentrations of both the activated indicator bacteria and the isolated probiotics were adjusted to 5 × 10^8^ CFU/mL. Pathogenic bacteria (100 µL) were evenly spread on MRS plates and allowed to dry for 3 min. Sterilized Oxford cups were then placed evenly on the plate surface (three wells per plate), and 100 µL of the isolated bacterial fluid was added to each Oxford cup. After incubating at 37 °C for 24 h, the diameter of the zone of inhibition was measured using a vernier caliper.

### 2.4. Whole-Genome Sequencing Analysis

The cell biomass of MH1 and ZJ1 was harvested after activation culture. Genomic DNA was extracted using the Wizard^®^ Genomic DNA Purification Kit (Promega, Madison, WI, USA) following the manufacturer’s protocol. The purified genomic DNA was quantified using a TBS-380 fluorometer (Turner BioSystems Inc., Sunnyvale, CA, USA). High-quality DNA was used for further analysis. Sequencing and bioinformatics analysis were conducted by Shanghai Majorbio Science and Technology Co., Ltd. using the Illumina NovaSeq6000 and Nanopore PromethION sequencing platforms. 

The raw Illumina sequencing reads generated from the paired-end library were subjected to quality filtering using fastp v0.23.0. The Nanopore reads were extracted, basecalled, demultiplexed, and trimmed using ONT Guppy with a minimum Q score cutoff of 7. The clean short and long reads were co-assembled to construct complete genomes using Unicycle v0.4.8. As a final step, Unicycler utilized Pilon v1.22 to polish the assembly using short-read alignments, reducing the rate of small errors. The genomic circles of MH1 and ZJ1 were plotted using Circos software version 0.69. The coding sequences (CDSs) of the chromosome and plasmid were predicted using Prodigal v2.6.3 and GeneMarkS, respectively. tRNA-scan-SE (v 2.0) was used for tRNA prediction, and Barrnap v0.9 was used for rRNA prediction. The predicted CDSs were annotated from NR, Swiss-Prot, Pfam, GO, COG, and KEGG databases using sequence alignment tools such as BLAST, Diamond, and HMMER. Additionally, the carbohydrate-active enzymes (CAZyme) in the genome were annotated and analyzed using the carbohydrate-active enzyme database (CAZy, http://www.cazy.org/ (accessed on 5 May 2022)).

### 2.5. Effect of Lactobacillus agilis MH1 and Lactobacillus salivarius ZJ1 on Mice

Activation and preparation of bacterial solution involved incubating *Lactobacillus agilis* MH1 and *Lactobacillus salivarius* ZJ1 with MRS medium at 37 °C for 24 h. The activated strains were then inoculated at 5% into MRS broth and incubated overnight. Subsequently, the strains were washed twice with sterile PBS and resuspended in PBS to achieve a viable bacteria concentration of 5 × 10^9^ CFU/mL, which was stored at 4 °C.

Mouse experimental design: forty male C57BL/6J mice (6-week-old) were obtained from Chengdu Gempharmatech Co., Ltd., Chengdu, China. The mice were provided with free access to water and food and were maintained under 12 h light/dark cycles at a temperature of 23 °C. After a week of acclimatization, the mice were randomly divided into four groups (*n* = 10): control group (PBS gavage); DSS group (PBS gavage + 3%DSS water); MH1 group (MH1 gavage + 3%DSS water); and ZJ1 group (ZJ1 gavage + 3%DSS water). Over a period of 15 days, the control and DSS groups were gavaged with sterile PBS, while the MH1 and ZJ1 groups were gavaged with *L. agilis* MH1 and *L. salivarius* ZJ1 solutions, respectively. Each animal received 200 µL of the respective solution per day for the designated time period. From day 8 to day 14, all mice were provided with purified water ad libitum. From day 15 to day 22, the treatment groups (DSS group, MH1 group, and ZJ1 group) received water containing 3% dextran sulfate sodium (DSS) [42], with the DSS water being changed every two days (Table S2). The acute enteritis model of mice was established by feeding 3%DSS water for 7–8 days. On day 23, the mice were anesthetized and sacrificed, and the necessary samples were collected for testing.

Determination of disease activity index (DAI): Throughout the experiment, the mice’s body weight, fecal status, and the presence of blood in the stool were weighed and recorded at the same time each day. The formulas for calculating weight change and disease activity index, as well as the scoring criteria for DAI, can be found in Table S3 [43]. The detection of blood in feces was performed using the Fecal Occult Blood Test Kit (Mlbio, Shanghai, China).

Colonic and spleen changes: After dissection, the colon and spleen were removed from the mice, and the length of the intact colon and the weight of the spleen were measured and photographed. The spleen index was calculated using the formula: spleen index (mg/g) = spleen weight (mg)/body weight (g).

Histological assessment: A 1–2 cm segment of the distal colon was fixed in a 4% paraformaldehyde solution for 48 h. The fixed colon tissues were then dehydrated, embedded in paraffin, sectioned into 5 µm thick slices, and stained with hematoxylin and eosin (H&E) for pathological analysis. The histological scoring criteria can be found in Table S4 [43].

Gene expression analysis: Total RNA was extracted from colon tissue using the Animal Total RNA Isolation Kit (Foregene, Chengdu, China), and its concentration was determined using the NanoDrop 2000 Spectrophotometer. Subsequently, the RNA was reverse-transcribed into cDNA using the reverse transcription kit (Vazyme, Nanjing, China). RT-PCR reactions were performed using the Real Time PCR EasyTM-SYBR Green I kit (Foregene, China). The β-actin gene of mice served as the endogenous control gene, and the specific primer sequences used for q-PCR are listed in Table S5. The relative expression levels of mRNAs (*IL-1β*, *IL-6*, *IL-10*, *TNF-α*, *Occludin*, and *ZO-1*) were calculated using the ΔΔCt method.

## 3. Results

### 3.1. Gut Microbial Profile of Native Chickens Based on 16S rRNA Sequencing

The collected native chicken samples were categorized according to different geographical environments and breeds within the same environment. Additionally, samples from different geographical environments were further divided into local chickens residing in typical high and low altitude regions, as well as local chickens from various areas with similar altitudes. We conducted 16S rRNA sequencing on the fecal microbiota of all samples. Following the quality control measures, a total of 8,432,881 effective sequences were obtained from 150 samples, averaging 56,219 sequences per sample (Table S6). Subsequently, we analyzed and compared the gut microbiota among different chicken groups.

### 3.2. Gut Microbial Diversity and Composition of Native Chickens at High and Low Altitudes

**Figure 2:**
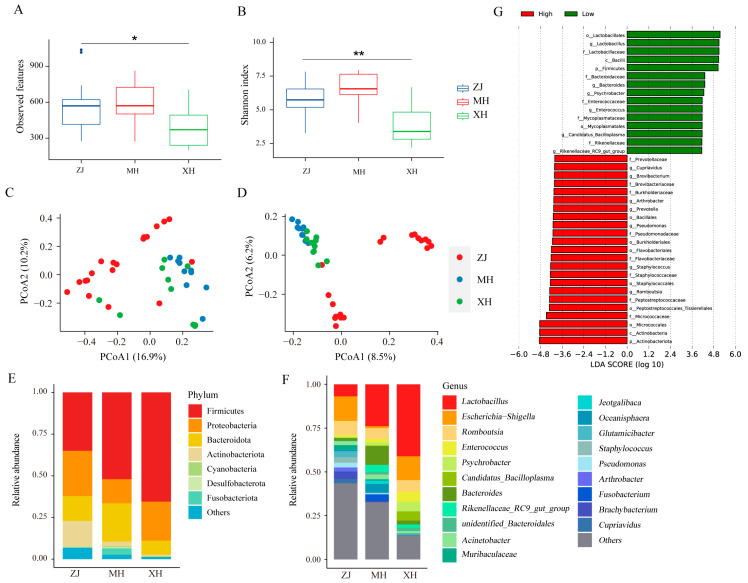
Microbial diversity and composition of native chickens at high and low altitudes. (**A**) Observed features index. (**B**) Shannon index. (**C**) Principal coordinate analysis (PCoA) of bacterial community structures using Bray–Curtis distance and (**D**) Jaccard distance. (**E**) The relative abundances of the top 7 phyla from fecal samples. (**F**) The relative abundances of the top 20 genera from fecal samples. (**G**) Biomarkers for different groups. * *p* < 0.05, ** *p* < 0.01.

To assess the alpha diversity of gut microbes at high and low altitudes, we calculated the observed features and Shannon index. The results indicated no significant difference between ZJ and MH, while the α-diversity of ZJ was significantly higher than that of XH (Figure 2A,B). Furthermore, principal coordinate analysis (PCoA) based on Bray–Curtis and Jaccard distances was employed to evaluate β-diversity. As depicted in Figure 2C,D, ZJ exhibited distinct separation from MH and XH, whereas MH and XH, representing chickens from low altitude, clustered together, implying a substantial dissimilarity in microbial composition between chickens at high and low altitudes. Further examination of the microbial composition at the phylum level (Figure 2E) revealed that Firmicutes, Proteobacteria, Bacteroidota, and Actinobacteriota were the predominant groups with higher abundances across all three groups. However, Firmicutes exhibited a higher abundance in the low-altitude group. At the genus level (Figure 2F), Lactobacillus exhibited a significantly higher abundance in the MH and XH groups compared to the ZJ group, whereas Escherichia-Shigella was the dominant group in ZJ. Notably, we identified unique microbiota at high and low altitudes, with Cupriavidus exclusively present in high-altitude chickens (ZJ), and Candidatus_Bacilloplasma exclusively present in low-altitude chickens (MH, XH). Subsequently, Lefse analysis identified a total of 38 significantly different bacterial taxa as biomarkers (Figure 2G), with 23 biomarkers associated with high altitude and 15 biomarkers associated with low altitude.

### 3.3. Microbial Profiles of Chickens at Similar Altitudes in Different Regions

**Figure 3:**
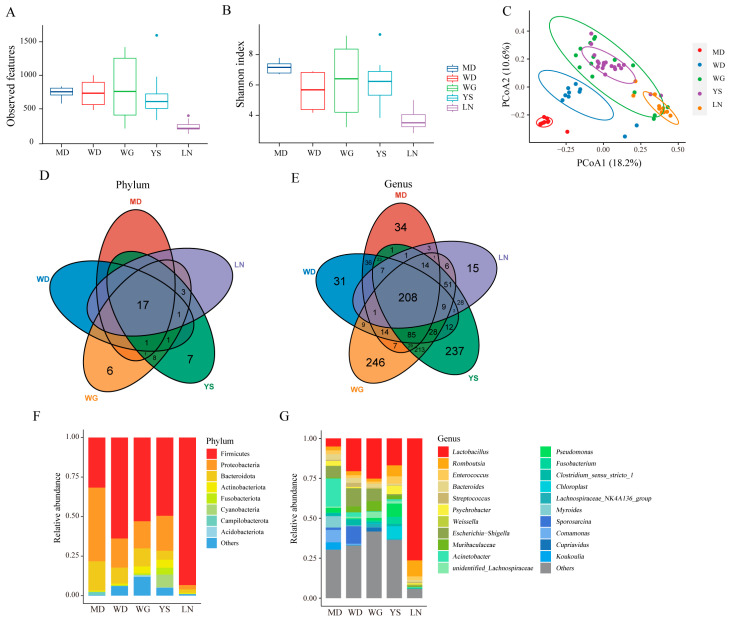
Variation of microbial diversity and composition in chickens from different regions. (**A**) Observed features index. (**B**) Shannon index. (**C**) Principal coordinate analysis (PCoA) of bacterial community structures based on Bray–Curtis distance. (**D**) Distribution of the microbial taxa showing the number of chickens in different areas at the phylum level and (**E**) at the genus level. (**F**) The relative abundances of the top 8 phyla from fecal samples. (**G**) The top 21 genera with the highest abundance.

We assessed the gut microbial diversity of chickens from different regions (MD, WD, WG, YS, and LN) with insignificant differences in altitude. Alpha diversity analysis indicated that MD exhibited the highest gut microbial diversity, while LN exhibited the lowest (Figure 3A,B). PCoA plots based on Bray–Curtis distances revealed that WG and YS clustered together, suggesting a similar microflora composition between these two groups, while the other groups exhibited distinct separation and significant differences (Figure 3C). To compare the differences in gut microbial composition and abundance among the five groups, we constructed a Venn diagram at the phylum and genus levels, resulting in a total of 50 phyla and 1616 genera. As shown in Figure 3D,E, there were 17 shared phyla and 208 shared genera among the five groups. Moreover, WG and YS exhibited six and seven unique phyla, respectively, while the remaining groups had none. However, each group possessed its own unique genera, with WG and YS harboring the highest number and LN exhibiting the lowest. This indicates that WG and YS have a more diverse unique gut microbiota compared to the other three groups. Further analysis of the microbial composition at the phylum level revealed that Firmicutes, Proteobacteria, and Bacteroidota were the dominant microbiota across all five groups, with the relative abundance of Firmicutes in LN reaching 93.36% (Figure 3F). At the genus level, MD exhibited the highest relative abundance of Acinetobacter, followed by Comamonas, Escherichia-Shigella, and Myrides. Lactobacillus showed the highest relative abundance in WD, WG, YS, and LN, with LN exhibiting a remarkable abundance of 76.36%. Additionally, Sporosarcina emerged as the dominant bacterium in WD (Figure 3G).

### 3.4. Microbial Profile of Commercialized Broiler and Laying Hens in the Same Farm

**Figure 4:**
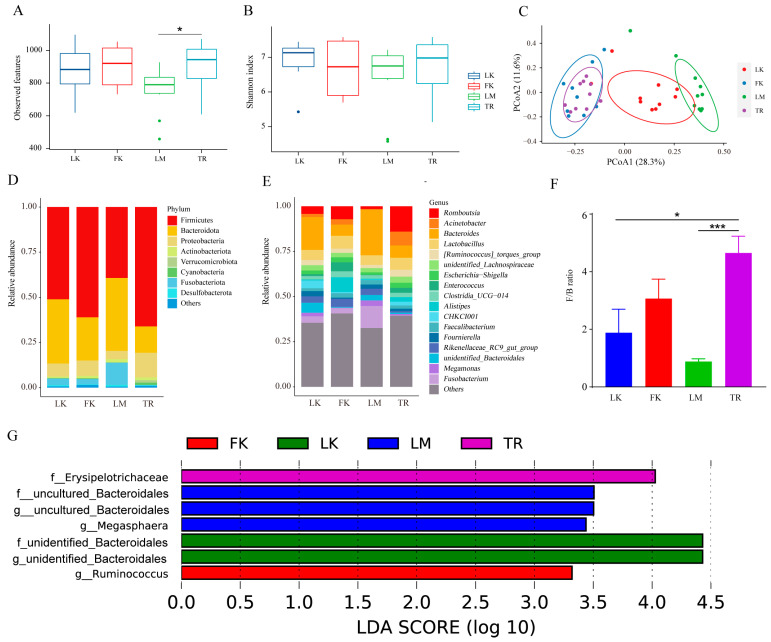
Changes in microbial diversity and composition in four chicken breeds. (**A**) Observed features index. (**B**) Shannon index. (**C**) Principal coordinate analysis (PCoA) of bacterial community structures based on Bray–Curtis distance. (**D**) The relative abundances of the top 8 phyla from fecal samples. (**E**) Seventeen genera with the highest relative abundance. (**F**) Firmicutes/Bacteroidetes (F/B) ratio. * *p* < 0.05, *** *p* < 0.001. (**G**) Significantly different microbiota from the four species.

The gut microbiota of chickens residing in distinct geographical environments exhibits significant variation, and within the same feeding environment, the gut microbiota of broilers and laying hens varies according to breed. We compared the α diversity of each group using the observed features and Shannon index. While no significant difference was observed in the Shannon index among the four groups, the observed features index of LM was significantly lower than that of TR (*p* < 0.05) (Figure 4A,B). We employed Bray–Curtis distance-based PCoA plots to assess the differences in gut microbiota composition among the four groups. Figure 4C illustrates that the gut microbiota composition of LK, LM, and TR exhibited significant dissimilarities, while the microbiota of FK showed relative similarity to that of TR. At the phylum level, Firmicutes, Bacteroidota, and Proteobacteria predominated in both broilers and layers. Notably, the abundance of Fusobacteriota was higher in the three laying hen breeds compared to TR, with Fusobacteriota accounting for the highest proportion in LM (12.23%) (Figure 4D). At the genus level, Romboutsia accounted for the highest proportion in TR (14.09%), Alistipes was the most abundant strain in FK (8.15%), and Fusobacterium emerged as the dominant strain in LM (12.23%) (Figure 4E). Furthermore, comparing the ratio of Firmicutes to Bacteroidetes (F/B) revealed the highest ratio in TR (Figure 4F). LEfSe analysis identified seven differentially abundant microbiota (LDA > 2) among the four breeds, as depicted in Figure 4G.

### 3.5. Isolation and Screening of Chicken-Derived Probiotics

**Figure 5:**
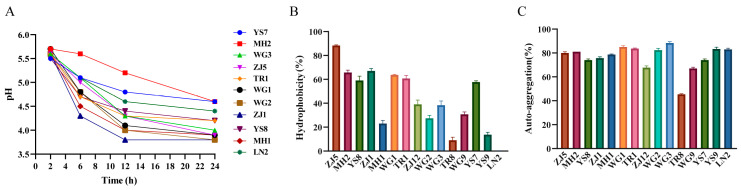
The results of the biological characteristics experiment of the isolated strains. (**A**) Acid production curve of isolated lactobacillus. (**B**) Surface hydrophobicity of chicken probiotics. (**C**) Auto-aggregation ability of chicken probiotics.

Probiotics play a crucial role in poultry production, and the development of chicken-derived probiotics, particularly among Chinese native chicken breeds with various advantages, holds significant importance. In this study, we isolated and identified a total of 15 strains from collected native chicken feces using selective media, including 11 strains of Lactobacillus and 4 strains of Bacillus (Table S7). We then examined the biological characteristics of these strains. The 24 h acid production test of Lactobacillus strains revealed a gradual decrease in pH value over time, stabilizing after 12 h. Notably, strains ZJ1 and MH1 exhibited the fastest decline rate and the lowest pH value after 24 h, indicating their strong acid production ability (Figure 5A). The Bacillus enzyme production assay demonstrated that only YS9 and ZJ12 could produce both protease and amylase (Table 1). Additionally, we evaluated the surface hydrophobicity and auto-aggregation ability of all strains. The results indicated that ZJ5, ZJ1, and MH2 displayed high hydrophobicity towards xylene, while most strains exhibited a high auto-aggregation ability (Figure 5B,C). Based on these results, we selected eight strains with superior performance for drug resistance testing. As shown in Table 2, Lactobacillus strains exhibited sensitivity or moderate sensitivity to most antibiotics, while Bacillus strains were sensitive to all antibiotics. Lastly, we evaluated the antimicrobial activity of the eight strains against *Escherichia coli*, *Staphylococcus aureus*, and *Salmonella*. The results demonstrated that ZJ1, TR1, and MH1 exhibited inhibitory activity against all three pathogens (Table 3).

### 3.6. Whole-Genome Analysis of MH1 and ZJ1

**Figure 6:**
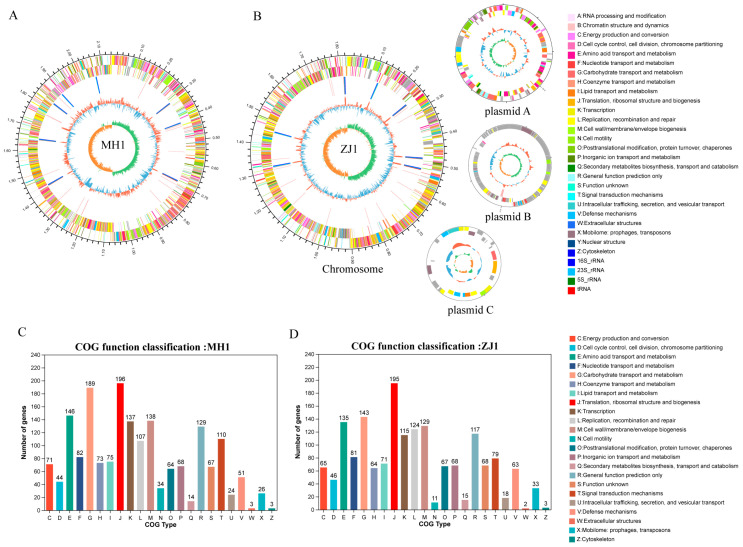
Genome features of Lactobacillus agilis MH1 and Lactobacillus salivarius ZJ1. (**A**) Circular genomic map of MH1 and (**B**) ZJ1. The two outermost circles are the CDS on the positive and negative chains, respectively, and the different colors indicate the functional classification of the different COGs of the CDS; the third circle is rRNA and tRNA; the fourth circle is GC content; The innermost circle is the GC skew value. (**C**) The COGs of the protein functional classification of the MH1 and (**D**) ZJ1.

Through screening, we selected two bacteria strains, MH1 and ZJ1, with superior probiotic performance, for a whole genome to explore their genomic profiles. The results of whole-genome sequencing showed that Lactobacillus agilis MH1 had a genome size of 2,142,805 bp with a GC content of 41.94%. The genome of MH1 comprised 1955 coding DNA sequences (CDSs), 90 tRNA genes, and 24 rRNA genes. Lactobacillus salivarius ZJ1 consisted of one chromosome and three plasmids, with a genome size of 2,147,960 bp and a GC content of 33.08%. ZJ1’s genome contained 2020 CDSs, 81 tRNA genes, and 22 rRNA genes (Table S8). The genome circle maps of MH1 and ZJ1 are depicted in Figure 6A,B, respectively. Furthermore, the CDSs of MH1 and ZJ1 were annotated using the COG database. In MH1, 1674 genes obtained COG gene annotation, while ZJ1 had 1547 annotated genes. These annotations were mainly associated with translation, ribosome structure, and biogenesis, carbohydrate transport and metabolism, and amino acid transport and metabolism (Figure 6C,D). Moreover, the comparison with the carbohydrate-active enzymes database led to the annotation of 84 carbohydrate-related enzymes in MH1 and 65 in ZJ1, with the majority annotated as glycoside hydrolases (GHs) and glycosyltransferases (GTs) (Table S9).

### 3.7. Alleviating the Effect of MH1 and ZJ1 on Colitis in Mice

**Figure 7:**
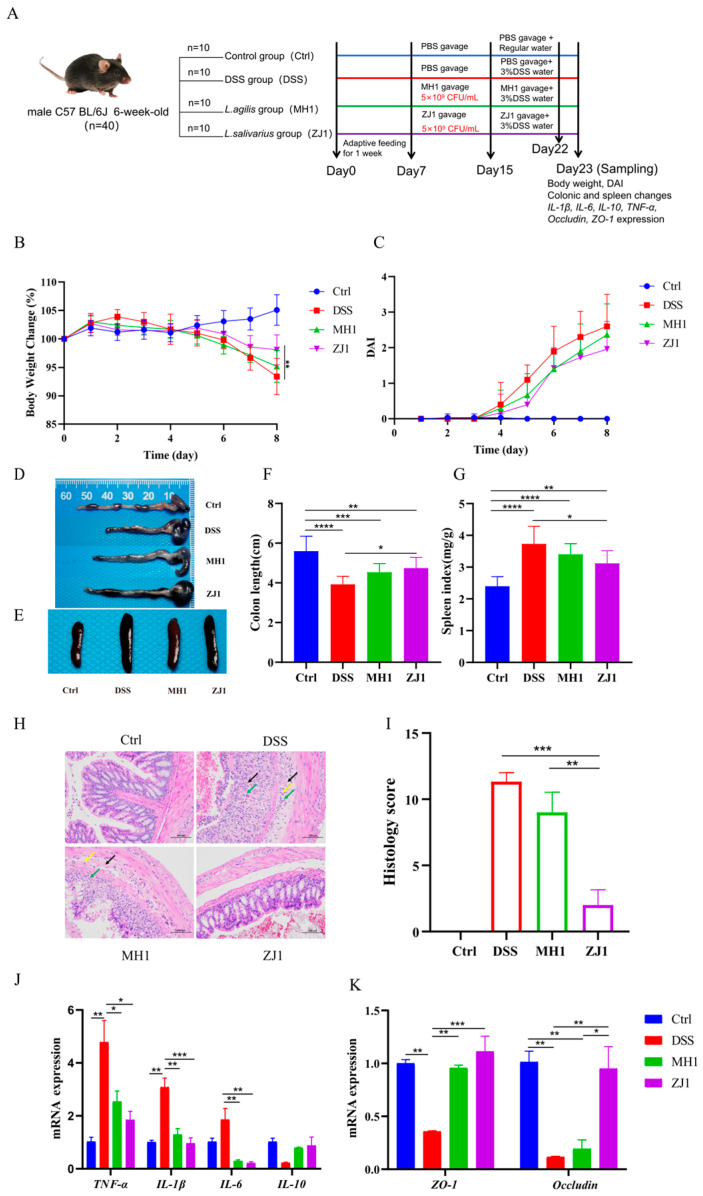
MH1 and ZJ1 alleviate the DSS-induced colitis in mice. (**A**) Overview of the workflow for integrated analysis in mice in four different groups. (**B**) Percentage change in body weight. (**C**) Disease activity index. (**D**) Colonic status of mice after DSS treatment. (**E**) Splenic status of mice. (**F**) Colon length in different groups of mice. (**G**) Spleen index in different groups of mice. (**H**) Pathological changes of colon in mice induced by colitis (H&E staining, 100×). The black arrow indicates the hyperplasia of connective tissue; the green arrow shows lymphocyte infiltration; the yellow arrow shows neutrophil infiltration. (**I**) Histological scoring of colonic pathology sections. (**J**) the mRNA expression levels of gut inflammatory factor and (**K**) tight junction protein in mice induced by colitis. * *p* < 0.05, ** *p* < 0.01, *** *p* < 0.001, **** *p* < 0.0001.

In order to investigate the immunomodulatory effects of MH1 and ZJ1 in animals, we established a DSS-induced colitis mouse model (Figure 7A). As shown in Figure 7B,C, the MH1 and ZJ1 groups exhibited less weight loss compared to the DSS group, and their disease activity index was also lower. The effect was more significant in the ZJ1 group than in the MH1 group. Additionally, we observed that the DSS model induced significant colon shortening and spleen enlargement, along with the obvious congestion and edema. However, the treatment with MH1 and ZJ1 resulted in a certain degree of recovery, alleviating these pathological injuries and reducing the spleen index (Figure 7D–G). Histologically, the gut tissue of the DSS group exhibited extensive ulceration, the invasion of injury into the submucosa, and the infiltration of inflammatory cells. After intervention with MH1 and ZJ1, the pathological symptoms of the colon tissue were alleviated. However, the remission effect was relatively poor in the MH1 group, with persistent connective tissue hyperplasia and a small amount of inflammatory cell infiltration observed in the colon tissue. Conversely, the histological morphology of the ZJ1 group resembled that of the control group, and the histological score was significantly lower than that of the DSS and MH1 groups (Figure 7H,I). At the molecular level, the DSS model significantly increased the mRNA expression levels of pro-inflammatory factors TNF-α, IL-β, and IL-6, while decreasing the mRNA expression level of the anti-inflammatory factor IL-10. However, after the intervention with MH1 and ZJ1, the expression of pro-inflammatory factors was significantly reduced, and the expression of anti-inflammatory factors was increased (Figure 7J). Similarly, the gut barrier function was impaired in mice with colitis, but the mRNA expression of tight-junction proteins ZO-1 and Occludin was increased in the MH1 and ZJ1 groups (Figure 7K).

## 4. Discussion

### 4.1. Gut Microbial Diversity of Chickens in Different Geographical Environments

The composition diversity of gut microbes plays a crucial role in the host’s environmental adaptability [22]. Tibetan chickens, residing in high-altitude harsh environments, exhibit unique gut microbial profiles. In our study, we analyzed the gut microbiota of ZJ at high altitude and MH and XH at low altitude. The results revealed significant differences in microbial diversity and composition between chickens at high and low altitudes, which aligns with the findings of Wu et al. [44] and Zeng et al. [45]. In terms of microbial composition, ZJ exhibited the highest abundance of *Escherichia-Shigella*, a Gram-negative Enterobacteriaceae family member known to cause diarrhea and dysentery. The adverse conditions and climate at high altitude may have facilitated the transmission of this bacterium [46]. The relative abundance of Firmicutes and *Lactobacillus* in chickens at low altitude was significantly higher compared to ZJ, with the proportions of these bacterial taxa decreasing with increasing altitude [47]. Moreover, *Cupriavidus* was unique to high-altitude chickens, while *Candidatus_Bacilloplasma* was exclusive to low-altitude chickens. *Cupriavidus* is abundant in *Glyptosternum maculatum* of the Qinghai–Tibet Plateau, exhibiting resistance to copper and high oxidative stress resistance [48,49,50]. In crayfish, *Candidatus_Bacilloplasma* plays a crucial role in the interaction between crayfish and microorganisms in the surrounding environment (water and sediment) [51]. This suggests that these unique microbes may contribute to environmental adaptation in chickens. 

Furthermore, we analyzed the gut microbiota of chickens from different regions at the same middle altitude. Previous studies have shown significant differences in gut microbiota among chickens from different regions or countries, with geographical location playing a dominant role in shaping the chicken gut microbiota [21,52]. Similarly, we observed differences in the gut microbial diversity and composition among the five regions in our study. Notably, LN from Xichang exhibited the lowest α diversity, with a significantly distinct microbiota composition compared to the other groups. Zhao et al. also reported a lower α diversity and distinct OTU composition in the gut microbiota of rhesus monkeys from Xichang compared to other geographical populations [53]. Xichang is characterized by a hot and dry valley area, and this difference may be attributed to its specific climatic conditions and food resources. Interestingly, LN had an extremely high relative abundance of Firmicutes and *Lactobacillus* in the gut. Firmicutes efficiently breaks down cellulose and lignin, while *Lactobacillus* plays a crucial role in food digestion and energy conversion [54,55,56]. On the other hand, WG and YS from Guizhou exhibited similar microbial composition, likely influenced by the complex and diverse forest vegetation and wide variety of plants in the region, leading to a more abundant presence of endemic microbes. 

### 4.2. Gut Microbial Diversity of Broiler and Laying Hens of Different Breeds

In our study, LM exhibited significantly lower microbial diversity compared to the other three local chicken breeds. This finding is consistent with Paul’s study, where the three native breeds in India displayed significantly higher microbial diversity and a number of OTUs compared to commercial broiler breeds [57]. Regarding bacterial composition, Firmicutes and Bacteroidetes were the dominant bacteria in all four species, with TR having the highest abundance and Firmicutes/Bacteroidetes ratio. The Firmicutes/Bacteroidetes ratio was associated with obesity and weight gain in livestock studies [58,59]. Bacteroides emerged as the dominant genus and biomarker. In poultry studies, Bacteroides has been positively correlated with cellulose digestibility in broiler feces, and a high abundance of Bacteroides has been linked to weight gain and feed conversion rate in chickens [60,61,62]. Additionally, Erysipelotrichaceae, *Ruminococcus*, and *Megasphaera* were identified as biomarkers for TR, FK, and LM, respectively. Erysipelotrichaceae is associated with the weight gain and is predominantly found in obese individuals [63]. The relative abundance of Erysipelotrichaceae was positively correlated with higher feed conversion, suggesting its role in nutrient digestion in chickens [64]. *Ruminococcus* is effective in fermenting cellulose, hemicellulose, and polysaccharides into acetate and succinate, while both *Ruminococcus* and *Megasphaera* contribute to gut health and influence host immune responses [65,66,67].

### 4.3. Isolation and Characterization of Chicken-Derived Probiotics

The gut microbiota of chickens has been recognized as an essential factor influencing their health, growth, and development, making it a promising source for probiotic research [12,68,69]. In this study, we isolated and characterized 11 strains of *Lactobacillus* and 4 strains of *Bacillus*. Lactic acid bacteria, such as *Lactobacillus*, produce lactic acid, which reduces the gut pH and inhibits the growth of pathogenic microorganisms, exhibiting prebiotic effects [70]. *Bacillus*, on the other hand, produces various digestive enzymes, including protease and amylase, which promote nutrient digestion and absorption in livestock and poultry [71,72]. Our findings revealed that MH1 and ZJ1 exhibited higher acid production capacity, while YS9 and ZJ12 showed higher enzyme production capacities. Additionally, we conducted screenings based on surface properties, drug resistance, and bacteriostatic tests. Surface properties, such as auto-aggregation and surface hydrophobicity, play a crucial role in the interaction between bacteria and the gastrointestinal mucosa, affecting their location and function within the gut [73]. Furthermore, probiotic strains should not serve as reservoirs for antibiotic resistance genes, posing a risk of transfer to gut pathogens [74]. Probiotics compete with pathogens for intestinal binding sites, reducing the colonization of pathogenic microbiota in the gut [75]. Finally, we selected two strains, MH1 and ZJ1, with an overall excellent performance and their whole genome profiles were consistent with previous studies [76,77,78]. Most of their coding genes are involved in carbohydrate transport and metabolism, indicating their strong carbohydrate utilization capacity and adaptability to various ecological niches [79]. Additionally, these strains are annotated to glycosyltransferases, which are essential for the formation of surface structures recognized by the host immune system [80].

### 4.4. Alleviative Effects of Lactobacillus agilis MH1 and Lactobacillus salivarius ZJ1 on Colitis

Pathogenic microbiota infestation or the dysbiosis of gut flora can impair the immune function of animals, leading to diarrhea and intestinal diseases with significant economic consequences in livestock and poultry breeding. Previous studies have demonstrated that certain *Lactobacillus* strains exhibit anti-inflammatory effects, such as *Lactobacillus plantarum*, which reduced intestinal inflammation and apoptosis induced by deoxylated valerianol in chickens [81]. *Lactobacillus reuteri* and *Lactobacillus johnsonii* showed efficacy in reducing the pathogen abundance and treating diarrhea in newborn calves [82]. In our study, we screened potential probiotics *Lactobacillus agilis* MH1 and *Lactobacillus salivarius* ZJ1. *Lactobacillus agilis* has been found to reduce the pathogenicity of enterotoxin-producing *Escherichia coli* and disrupt the structural integrity and protein synthesis of *Escherichia coli* [83]. Studies have also demonstrated the potential antioxidative, anti-inflammatory, and anti-diabetic properties of *Lactobacillus agilis* isolated from plants [84]. *Lactobacillus salivarius* has been shown to alleviate liver injury through the miR-130a-5p/MBOAT2 signaling pathway, regulate gut microbiota, reduce proinflammatory cytokines, and repair the intestinal barrier, thereby improving intestinal mucositis [85,86]. In our study, using a mouse model of intestinal inflammation induced by DSS, we found that MH1 and ZJ1 exerted a protective effect against intestinal inflammation.

## 5. Conclusions

The gut microbiota of chickens residing at different altitudes and in various regions exhibit significant differences, and each has its unique microbiota, reflecting the adaptability of native chickens to their respective environments. Similarly, there are variations in gut microbial diversity and structure among different breeds of broilers and laying hens reared in the same environment. The dominant bacterial taxa in broilers and laying hens include Firmicutes, Bacteroidota, and Proteobacteria, with distinct biomarkers identified for each breed. Additionally, we successfully isolated and characterized chicken-derived probiotics, namely *Lactobacillus agilis* MH1 and *Lactobacillus salivarius* ZJ1, and obtained their genomic profiles. Furthermore, our research demonstrated that MH1 and ZJ1 possess the ability to alleviate DSS-induced colitis in mice and exhibit immune regulatory functions.

## Figures and Tables

**Table 1:** Results of enzyme production of Bacillus from chicken.

Strains	Protease (H/C)	Amylase (H/C)
TR8	1.43	-
YS9	1.73	1.64
ZJ12	1.55	1.95
WG9	-	-

Enzyme production capacity = diameter of hydrolysis circle H (mm)/diameter of colony C (mm).

**Table 2:** Results of the sensitivity of chicken probiotics to different drugs.

Antibiotics	ZJ1	ZJ5	WG1	WG3	TR1	MH1	YS9	ZJ12
Ampicillin	S	S	S	S	S	S	S	S
Cefotaxime	S	S	S	S	S	S	S	S
Penicillin	S	S	S	S	R	S	S	S
Amikacin	R	I	R	R	S	R	S	S
Streptomycin	R	S	S	S	R	R	S	S
Gentamicin	I	R	R	I	S	R	S	S
Norfloxacin	I	R	R	R	R	I	S	S
Ciprofloxacin	I	R	R	R	R	I	S	S
Levofloxacin	I	R	R	R	R	I	S	S
Erythromycin	S	S	S	S	S	R	S	S
Tetracycline	R	S	R	R	R	R	S	S
Vancomycin	R	S	S	S	R	R	S	S
Chloramphenicol	S	S	S	S	S	S	S	S
Clindamycin	S	I	I	R	I	R	S	S

S, sensitive; I, intermediate; R, resistant.

**Table 3:** Bacteriostatic test results of chicken probiotics.

Strains	Diameter of Inhibition Zone (mm)		
	*Escherichia coli*	*Staphylococcus aureus*	*Salmonella enteritidis*
ZJ1	24.42 ± 0.40	15.87 ± 0.73	30 ± 0.49
ZJ5	13.05 ± 0.34	-	17.86 ± 0.34
WG1	12.52 ± 0.46	-	24.1 ± 3.32
WG3	13.88 ± 0.79	-	19.61 ± 0.37
TR1	18.36 ± 0.42	11.03 ± 0.44	23.35 ± 0.51
MH1	17.68 ± 0.59	9.49 ± 0.40	28.47 ± 0.25
YS9	-	-	-
ZJ12	-	-	-

## Data Availability

The datasets presented in this study can be found in online repositories. The names of the repository and accession number(s) are as follows: PRJNA995374.

## Supplementary Materials

The following supporting information can be downloaded at: https://www.mdpi.com/article/10.3390/ani13233672/s1, Table S1: Sampling information in different areas; Table S2: Experimental design of mice; Table S3: Scoring criteria of disease activity index (DAI) in mice; Table S4: Colon histological injury standard score; Table S5: The primer information of qPCR; Table S6: Summary statistics of 16S rRNA gene sequencing from 150 samples; Table S7: Isolation and identification results of chicken probiotics; Table S8: Basic genomic characteristics of MH1 and ZJ1 strains; Table S9: Annotated results of *Lactobacillus agilis* MH1 and *Lactobacillus salivarius* ZJ1 CAZy database.
